# Which tree orders in southern Africa have the highest antimicrobial activity and selectivity against bacterial and fungal pathogens of animals?

**DOI:** 10.1186/1472-6882-14-317

**Published:** 2014-08-27

**Authors:** Elisabeth Pauw, Jacobus Nicolaas Eloff

**Affiliations:** Phytomedicine Programme, Department Paraclinical Sciences, Faculty Veterinary Science, University of Pretoria, Private Bag X04, Onderstepoort, 0110 South Africa

## Abstract

**Background:**

The study randomly screened leaf extracts of several hundred southern African tree species against important microbial pathogens to determine which taxa have the highest activity and may yield useful products to treat infections in the animal health market.

**Methods:**

We determined the antibacterial and antifungal activity of 714 acetone leaf extracts of 537 different tree species against *Enterococcus faecalis*, *Staphylococcus aureus*, *Escherichia coli*, *Pseudomonas aeruginosa, Candida albicans* and *Cryptococcus neoformans*. A sensitive serial dilution microplate method was used.

**Results:**

Several extracts had MICs as low as 0.02 mg/ml. We analysed 14 out of the 38 tree orders where we determined the activity of more than 8 different tree species representing 89% of all species examined. There were statistically significant differences in some cases. Celastrales, Rosales and Myrtales had the highest activity against Gram-positive bacteria, the Myrtales and Fabales against the Gram-negative bacteria and the Malvales and Proteales against the fungi. Species present in the Asterales followed by the Gentiales and Lamiales had the lowest activities against all the microorganisms tested. Fabales species had the highest activities against all the microorganisms tested. There was substantial selectivity in some orders. Proteales species had very high activity against the fungi but very low activity against the bacteria. The species in the Celastrales and Rosales had very low antifungal activity, low activity against Gram-negative bacteria and very high activity against Gram-positive bacteria.

**Conclusion:**

Against all classes of microorganisms, the four orders containing species with the highest average antimicrobial activities also contained several species with low activities against different pathogens and *vice versa*. These results therefore should be used with circumspection in selecting tree orders that would yield the highest probability of finding species with promising activities. Nevertheless there was a twofold increase in probability of finding extracts with interesting antifungal activity from orders with high mean activity than from orders with low mean activity. The probability increased to threefold and fivefold for Gram-positive and Gram-negative bacteria respectively.

## Background

Infections are the world’s leading cause of premature deaths, killing almost 50 000 people every day [1]. The pharmacological industry produces a large amount of antibiotics but the extensive and inappropriate use of antibacterial and antifungal agents led to a significant upsurge in resistance to these drugs [2, 3]. The treatment of bacterial and fungal pathogens that are drug-resistant is even more complicated in acquired immune deficiency syndrome (AIDS) patients [4]. These developments have increased the need to search for new antibacterial products with improved activity [5].

Plants produce a diverse range of bioactive molecules with a wide spectrum of activities, making them a rich source of different types of compounds that could be used as medicine [6, 7]. Throughout the world, plants are used to treat many illnesses, particularly infectious diseases, and were once used as the primary medicines all over the world [8, 9]. There has been growing world-wide interest in natural and traditional medicines as an alternative form to treat infectious diseases [8]. This is partially based on the widely held, but not necessarily correct assumption, that natural medicine is safer. The World Health Organisation (WHO) estimated that about 80% of the rural populations of the developing countries rely exclusively on plants to meet their health care needs [10]. Nevertheless, of all the c. 250 000 species of higher plants on earth, only a fraction has been examined for all aspects of their potential therapeutic medicinal value [11]. Furthermore, natural products and their derivatives (including those from microorganisms) represent more than 50% of all drugs in clinical use in the world [12]. The importance of natural products in drug discovery has been discussed in many scientific papers [13].

Southern Africa has a rich floral diversity comprising in the order of 10% of the world’s plant diversity on less than 2.5% of Earth’s land surface [14]. According to Goldblatt [15] about 80% of the plants in the Western Cape are endemic to this region. This diversity represents a very valuable resource for commercial development as well as basic scientific studies [16]. In South Africa in particular, many rural ethnic groups rely on traditional indigenous plant knowledge to treat various diseases in both humans and livestock [17, 18]. In the order of 15% of the 24 000 taxa recorded in southern Africa are used in traditional medicines [19, 20] and an estimated 500 plant species are traded in informal medicinal plant markets [21]. Traditional medicine remains more affordable than Western medicine and is also easily accessible by the poorer communities.

In recent years, the rate of information documented on the biological activity and chemistry of plants used in traditional medicine in southern Africa have increased [22] and several studies provided scientific support for the use of various African plants for treating infections and diseases [23]. Several southern African medicinal plants have been identified in the first African Herbal Pharmacopoeia [24].

Contrary to expectations, thousands of publications on antimicrobial activity of plant extracts have not led to the development of any new commercial antimicrobial compounds of significant importance world-wide [25]. Many large-scale screening programmes of the past failed to produce worthwhile plant-derived antimicrobial pharmaceutical products [5, 26]. Consequently, the major pharmaceutical companies have lost interest in screening higher plants for their biological potential [27].

Some reasons for the failure of these screening programmes could be:

- methods such as agar diffusion that are inaccurate and misleading for plant extracts have been used by many researchers [5],
- many scientists following traditional healers, used water while it has been shown that the antimicrobial compounds are usually intermediate polarity compounds that are not extracted by water [28, 29],
- several publications considered excessively high MIC’s (>5 mg/ml) as active [25, 30–32]
- many scientists selected plant species for research studies based on traditional knowledge [5].

Traditional healers typically use aqueous extracts of plants which, as stated above, generally have very low activities [28, 33]. Water does not extract the antimicrobial compounds that usually have an intermediate or non-polar character [29]. The activity of effective aqueous extracts used by traditional healers may be based on an indirect effect by stimulating the immune system of the host rather than killing the pathogens. Aqueous extracts of plants species used in ethnomedicine may therefore not have high direct antimicrobial activity and scientists may consequently have focussed on the wrong species.

Moreover, many scientists focussed on the isolation of compounds not recognising that phytomedicines contain a mixture of compounds that often acts synergistically [12]. In our experience antimicrobial compounds isolated from extracts never had the expected activity based on the activity of crude extracts and fractions [34]. This is probably because different plant metabolites may work in combination with other compounds to regulate microbial infections and may therefore not be effective alone [26].

For these reasons coupled with the large number of plant species that have not yet been examined for their antimicrobial activities and the urgent need to discover new antimicrobial agents, we decided to screen southern African plant species to identify promising antimicrobial plant extracts against six important pathogens as leads for further in-depth research. Such an approach has not been followed in southern Africa before, except partially in the work of Noristan Pharmaceuticals [35]. The methods they used to determine antimicrobial activity are now outdated, thereby rendering comparisons impossible. Our focus was to sample leaves of representatives of southern African tree genera and families for ease of collection and sustainable use in case exciting activities are found. We used a standardised method for analysing the antimicrobial activities. A wide-screening approach offered the potential to discover plants with antimicrobial activities that are available in rural areas but which are not used traditionally. It also reduced the administrative complications of investigating plants based on its use in traditional medicine due to the new legislation to prevent biopiracy and promote benefit sharing in South Africa [5].

This study was part of a preliminary extensive screening programme of the Phytomedicine Programme, Department Paraclinical Sciences, University of Pretoria. The main aim of the screening was to facilitate the discovery of tree leaf extracts with high activities that may yield useful products for the herbal medicine that can be used to combat microbial infections in animals and humans. Preliminary data obtained in this study provided material for several masters and doctorate projects for students. Patents have been filed on some of the results.

The secondary aim of the screening study was to compare the antimicrobial activities of several southern African tree species at two taxonomic levels: suprafamilial (order) and suprageneric (family) to investigate if some orders and families generally contained tree species with higher antimicrobial activities compared to other taxa. Further studies could then focus on related taxa in promising orders and families to facilitate and improve the selection process. This approach may facilitate and optimise the selection of tree species for the discovery of new antimicrobial plant extracts by saving time and cost.

Given that related taxa may contain similar or related pharmacologically active compounds and therefore similar bioactivity [36, 37], the correlation between taxonomy and antimicrobial activity was investigated. The rationale was that taxa with general high activity could offer more promising leads. If good correlations were found, it could lead to a better guided approach in selecting tree species from promising families for continuing studies rather than based on ethnopharmacology. This information could also be useful for related taxa of plants growing outside South Africa. A number of closely related taxa, assumed to contain related active compounds could then be screened.

In this paper we compare the antimicrobial activities of tree extracts at order level based on the average minimum inhibitory concentration (MIC) of all the species that we have analysed within each order. Since orders are more inclusive compared to families, the mean MIC’s calculated for each of the orders should therefore be less affected by outliers. In addition, since the boundaries of orders are more accepted, a comparison at this level may reduce variation caused by changes in, or differences between classification systems. We also compared the antimicrobial activities of the species present in the four orders with the highest activities with the species present in the four orders with the lowest activities to determine the practical value of the differences between orders with high and low activity.

## Methods

### Study area and study material

The study covered the floristic area of southern Africa, south of the Kunene, Okavango and Zambezi rivers and includes the countries of South Africa, Lesotho, Swaziland, Namibia, Botswana, Zimbabwe and a part of Mozambique. This is a large area, covering several climatic zones and a wide range of vegetation types [38, 39]. An estimated 24 000 higher plant taxa of 368 families are recorded in the Flora of Southern Africa [14].

It was practically impossible to screen all the 24 000 southern African plant species. In order to reduce this project to more feasible dimensions the study focussed on tree species because identification of trees is easier and can be done with a higher degree of certainty. We sampled leaf material only because leaves are a renewable resource and it is also easier to recollect leaves from the same plant for follow-up work. Finally, if a product is to be developed from the plant material, leaves of trees will be a sustainable resource.

### Plant collection

Leaf samples of southern African trees were nearly exclusively collected in National Botanical Gardens of the South African National Biodiversity Institute (SANBI) across South Africa: Pretoria National Botanical Garden (Pretoria), Lowveld National Botanical Garden (Nelspruit), KwaZulu-Natal National Botanical Garden (Durban), Kirstenbosch National Botanical Garden (Cape Town) and the Harold Porter National Botanical Garden (Betty’s Bay). We also collected leaves from the Manie van der Schijff Botanical Garden and the Onderstepoort Poisonous Plant Garden at the University of Pretoria.

These gardens were selected based on easy access to facilitate the recollection of leaves from the same tree for future collections to expand the work. Several collection trips were undertaken between May 2004 and March 2009 to collect the leaf material. The identification of the leaf material was verified by the respective botanical garden herbariums. In the botanical gardens most trees were labelled and voucher specimens of the trees along with collection data and origin of collection are kept in the respective National Botanical Garden herbaria. GPS-coordinates were recorded for unlabelled trees. In all cases voucher specimens are also stored in the HGW Schweickert Herbarium of the University of Pretoria.

Approximately 2 100 tree species are found in the southern African region [39] which includes a few marginal (shrub-like) tree species as well as woody climbers. To screen representative samples of all 2 100 species was not practical due to time and financial restrictions as well as the limited number of trees present in botanical gardens. Our target was therefore to collect good representative members of at least one species per genus in a family. However, we sampled more than one species from the larger genera. This approach reduced the number of species to a more manageable size.

For practical reasons, we sampled only in South Africa and samples of genera occurring outside the borders of South Africa were only collected if the relevant representative tree species were found in one of the National Botanical Gardens. Therefore, a large proportion of tree genera distributed outside the South African borders (32%) were not collected. These factors resulted in orders, especially those with a limited number of genera and families, being incompletely represented. The orders Huerteales, Piperales, Poales, Ranunculales, Solanales and Welwitchiales were not represented.

In total, 717 samples of 537 tree species spanning 350 genera, 101 families and 38 orders were collected. The collection of samples that provided the leaf material for the study has been labelled the Phytomedicine Tree Database (PMDB), Paraclinical Sciences, Faculty of Veterinary Sciences, University of Pretoria. A detailed list of the species, genera, families and orders collected is available from the Phytomedicine Programme (see http://www.up.ac.za/phyto).

### Taxonomical arrangement of the southern African tree species

The 2 100 trees of southern Africa are placed into five subclasses: Polypodiidae, Cycadidae, Gnetidae, Pinidae (tree ferns and gymnosperms) and Magnoliidae (angiosperms) [40]. Grouping of the trees of southern Africa resulted in a total of 44 orders. Plant classification results in an unequal distribution of representative species per family and order. Consequently some plant families and orders are very large containing several genera and species, while others contain only one or a few species. In addition, the boundaries of this study (trees of southern Africa) moderated the size of the families and orders. Although orders are in general more inclusive than families, several of the orders contained only one or two families which in turn contained only a few genera or in some cases a single genus.

The 44 orders representing the tree species in southern Africa contained from 1 to 77 genera per order [39, 41]. The largest tree orders were Gentianales (77 genera), Fabales (76 genera), Malpigiales (74 genera) and Sapindales (56 genera). In addition to size differences, we found that the range of order sizes was extremely skewed with far more small than large orders. Thirteen of the 44 orders contained only a single southern African tree genus while 17 orders contained between two and 8 genera and 14 orders contained 9 or more genera (Table 1). Only five of the orders contained 30 or more genera.

**Table 1:** **Summary of the orders encompassing the southern African tree species analysed for the Phytomedicine tree database (PMDB)**

Number of tree species analysed per order	Number of orders	Orders
1	11	Aquifoliales, Bruniales, Canellales, Caryophyllales, Cornales, Crossomotales, Cyatheales, Fagales, Oxalidales, Pandanales, Zygophyllales
2 to 8	13	Arecales, Asparagales, Brassicales, Buxales, Coniferales, Cycadales, Geraniales, Laurales, Pinales, Santalales, Saxifragales, Vitales, Zingiberales
≥ 9	14	Apiales, Asterales, Celastrales, Ericales, Fabales, Gentianales, Lamiales, Magnoliales, Malpighiales, Malvales, Myrtales, Proteales, Rosales, Sapindales

### Plant preparation

Harvested leaves were immediately stored in open mesh loosely woven bags to ensure air flow for quick drying and to minimise chemical changes by microbial attack after collection. The leaf material was examined and any leaves attacked by insects or microbes were removed.

The leaves were dried indoors at room temperature under good ventilation conditions and, when completely dried, ground to a fine powder using a Jankel and Künkel Model A10 mill. The powder was stored in tightly closed glass containers in the dark at room temperature. Dried material was used because there are fewer problems associated with large scale extraction of dried plant material compared to fresh plant material [42] and dried material may retain its biological activity for many decades [43].

### Extraction method

Acetone (technical grade, Merck) was used as an extractant in the assays using a ratio of 1:10 of leaf material to extractant. Eloff [44] found that acetone was the best choice as an extractant mainly due to its ability to extract compounds of a wide range of polarities [28], its non-toxicity to bioassay systems [45] and because it is easy to remove it from extracts.

The extraction procedure developed and described by Eloff [44] was used. Three gram (3.0 g) of each tree leaf sample was extracted with 30 ml acetone. The mixture was shaken at high speed for 10 minutes in a Labotec model 20.2 shaking machine. The extracts were then centrifuged at 6000 rpm for 10 minutes. After centrifugation, the supernatants were filtered through Whatman No 1 filter paper and transferred into pre-weighed labelled glass vials. The solvent was removed under a stream of air at room temperature for quantitative determination and dissolved in acetone to a concentration of 10 mg/ml.

### Microbial test organisms

The panel of microbial organisms used in this study represented pathogenic species of different classes commonly associated with nosocomial infections.

The bacteria were maintained in the Phytomedicine Laboratory at Onderstepoort, University of Pretoria and consisted of two Gram-positive strains, *Enterococcus faecalis* (ATTC 29212) and *Staphylococcus aureus* (ATTC 29213), and two Gram-negative strains, *Escherichia coli* (ATTC 25922) and *Pseudomonas aeruginosa* (ATTC 27853). The specific bacterial strains were recommended by the National Committee for Clinical Laboratory Standards [46]. All the bacterial strains were subcultured from the original strains, stored at −70°C and maintained on Müller Hinton (MH) agar plates at 4°C. Three to five colonies of bacteria from a fresh 18–24 h agar plate culture were inoculated into 2 ml of sterile distilled water with 0.02% Tween 80 (BDH). From this mixture, 1–10 μl were transferred to 10 ml MH broth to give a final concentration of approximately 5 × 10^5^ CFU/ml.

Two of the most common and important disease-causing fungi in animals, *Candida albicans* and *Cryptococcus neoformans* [47] were used. The fungal organisms were maintained in the Microbiology Laboratory at Onderstepoort, University of Pretoria. The test organisms, both yeasts, were cultured from clinical cases of disease in animals at the Department of Veterinary Tropical Diseases, Faculty of Veterinary Sciences, University of Pretoria. None of the animals was treated prior to sampling. All fungal strains were maintained on Sabouraud dextrose agar (Oxoid, Basingstoke, UK). Sabouraud Dextrose broth was used as liquid nutrient medium.

### Microdilution assays

The minimum inhibitory concentration (MIC) of the plant extracts was determined in triplicate for each assay. The MIC was expressed as the lowest concentration of the extract that led to a decrease in microbial growth.

#### Antibacterial microdilution assay

A widely accepted sensitive serial dilution microplate method [48] was used to determine the minimum inhibitory concentration (MIC) of plant extracts against four bacterial strains in triplicate. This biological assay was chosen because of its simplicity, reproducibility, sensitivity, and relatively low cost while being a rapid method at the same time. The dried extracts were dissolved in acetone to a concentration of 10 mg/ml and 100 μl was added to the first well of a 96 well microtitre plate and were serially diluted 1:1 with water. Overnight incubated bacterial cultures (100 μl) were added to each well. Starting with 10 mg/ml, the bacteria were therefore subjected to final concentrations of 2.50 mg/ml to 0.02 mg/ml. The 50% inoculum of microorganisms in the logarithmic growth phase means that a minor contamination would not influence the results. Gentamicin (100 μl of 0.1 mg/ml) was used as positive control and acetone was used as solvent control. The microplates were incubated overnight at 37°C in 100% relative humidity.

As an indicator of growth, 40 μl of 0.2 mg/ml INT (*p*-iodonitrotetrazolium violet, Sigma®) dissolved in hot water was added to the microplate wells and incubated at 37°C for 2 hours. The MIC was determined visually after 2 hours.

#### Antifungal microdilution assay

For the antifungal assay, the sensitive serial dilution microplate method described by [48], modified by [49], was used. Two-fold serial dilutions of extracts were prepared as described for the bacteria. Actively growing fungal organisms were transferred from an agar plate by collecting conidia with a sterile cotton swab into a fresh Sabouraud Dextrose broth and 100 μl of this mixture was then added to each well as described for the bacteria in the previous section. Amphotericin B was used as positive control and acetone was used as solvent control. As an indicator of growth, 40 μl of 0.2 mg/ml INT (*p*-iodonitrotetrazolium violet, Sigma®) dissolved in water was added to the microplate wells and the plates were incubated at 35°C for 24 hours after which the MIC was determined.

### Data processing

#### Grouping and size scale of the orders

The order level grouping of the tree species of southern Africa belonging to the subclass Magnoliidae (angiosperms) followed the APG III classification system [50] while those belonging to the subclasses Polypodiidae, Cycadidae, Gnetidae and Pinidae (tree ferns and gymnosperms) were grouped into orders following the SANBI (South African National Biodiversity Institute) Internet Taxonomy System of the Trees of southern Africa.

#### Order level comparison of the minimum inhibitory concentrations (MIC)

In total 717 crude extracts of 537 tree species were screened. Mean MIC values in this study were presented as a geometric mean which normalises the averaged values so that no single value dominates the weighting. All calculations were done using Microsoft Excel version 2010. For the purpose of the calculation of mean MIC’s, a value of 0.02 mg/ml was assigned for the extracts with an MIC lower than 0.02 mg/ml and for the few extracts with an MIC higher than 2.50 mg/ml, a value of 2.50 mg/ml was assigned. A geometric mean MIC was determined for each of the tree species after which the species were grouped into their respective orders and a mean MIC value was calculated for each of the orders against *E. faecalis, S. aureus, E. coli*, *P. aeruginosa, C. albicans* and *C. neoformans.* A geometric mean MIC was also calculated for each order against each of the three pathogen classes: Gram-positive bacteria (*S. aureus* and *E. faecalis*), Gram-negative bacteria (*E. coli* and *P. aeruginosa*) and fungi (*C. albicans* and *C. neoformans*).

#### Statistical analysis

To compare the different orders, the geometric mean MIC values calculated for each order against the three pathogen classes, were log transformed after which an ANOVA and a *post-hoc* test (Least square means) were performed. Statistical analysis were performed using the statistical software program SAS (version 9.2). Statistical significance was defined at p < 0.05 level. In cases where statistical significances were established, the practical significance of differences was challenged, in accordance with the recommendations of [51].

#### Antimicrobial activities of extracts of tree species within the orders with highest and lowest activities

To determine the practical value of the differences between orders with high and low activity we grouped the MICs in the following categories: < 0.03 mg/ml; 0.03 to 0.04 mg/ml; 0.05 to 0.08 mg/ml; 0.09 to 0.16 mg/ml; 0.17 to 0.31 mg/ml; 0.32 to 0.63 mg/ml; 0.64 to 1.25 mg/ml and 1.26 to 2.50 mg/ml. The antimicrobial activities of the species present in the four orders with the highest activities were compared with the antimicrobial activities of the species present in the four orders with the lowest activities. This was done separately for each class of microorganism.

## Results

### Grouping and size scale of the orders

We analysed 537 tree species of southern Africa. Those species are distributed in 38 tree orders and the number of species per order ranged from 1 to 71. The orders with the largest number of tree species captured were Malpighiales (71 tree species), Sapindales (64 tree species), Gentianales (64 tree species) and Fabales (57 tree species). Twenty four of the 38 orders in the database were represented by eight or less species (Table 1).

### Antibacterial activities of orders

The mean activities of the orders for which we examined less than nine tree species were not analysed statistically because of the small sampling number. Therefore, the mean antimicrobial activities of only the 14 orders of which we analysed nine or more species are presented in this paper. These 14 orders did however contain a majority (89%, 476 out of 537) of all the tree species we analysed.

The orders had different levels of activity against each of the pathogens. In the respective tables, mean MIC’s against each of the pathogens as well as a mean MIC for each pathogen class were listed. The mean MIC against the pathogen class was used to compare the orders.

The differences between the mean MIC values against the Gram-positive bacteria of the 14 largest orders ranged between 0.30 and 0.80 mg/ml (Table 2). An ANOVA revealed significant differences (p < 0.05) between the mean MIC of the orders. The orders with the highest activity against Gram-positive bacteria were Celastrales, Rosales and Myrtales, with a mean MIC of 0.30 mg/ml. A *post-hoc* least square mean test (LSM) established that these three orders had significantly higher activities compared to the five orders with the lowest activities. Furthermore, the extracts of species in the orders Fabales, Ericales, Sapindales and Malpighiales were significantly more active compared to Asterales, the order with the lowest mean activity. Species in the orders Asterales, Magnoliales, Gentianales, Lamiales and Proteales had relatively low mean activities. The Asterales had a significantly lower mean activity (p < 0.05) compared to the seven orders with the highest activities while the Magnoliales, Gentianales, Lamiales and Proteales had significantly lower mean activities (p < 0.05) than that of the Celastrales, Rosales and Myrtales. With a few exceptions there were not major differences in the mean activities of the orders against the two Gram-positive bacteria (Table 2).

**Table 2:** **Mean minimum inhibitory concentrations (MIC) ± standard deviation (SD) of species present in the different orders against the Gram-positive bacteria**

Order	n (≥9)	MIC (mg/ml) (±SD)		
		***E. faecalis***	***S. aureus***	Mean of Gram-positive bacteria
Celastrales	19	0.23 ± 0.41	0.37 ± 0.69	0.30 ± 0.47^a^
Rosales	28	0.21 ± 0.35	0.41 ± 1.04	0.30 ± 0.66^a^
Myrtales	25	0.28 ± 0.74	0.31 ± 0.52	0.30 ± 0.53^a^
Fabales	57	0.38 ± 0.79	0.41 ± 0.92	0.40 ± 0.81^ab^
Ericales	30	0.28 ± 0.47	0.56 ± 1.10	0.40 ± 0.72^ab^
Sapindales	64	0.34 ± 0.88	0.52 ± 1.04	0.42 ± 0.92^ab^
Malpighiales	71	0.38 ± 0.84	0.53 ± 1.03	0.45 ± 0.86^ab^
Malvales	26	0.34 ± 0.57	0.65 ± 0.93	0.47 ± 0.67^abc^
Apiales	9	0.44 ± 0.71	0.59 ± 1.04	0.51 ± 0.75^abc^
Proteales	28	0.38 ± 0.49	0.71 ± 1.05	0.52 ± 0.73^bc^
Lamiales	35	0.44 ± 0.62	0.63 ± 1.00	0.53 ± 0.72^bc^
Gentianales	64	0.41 ± 0.67	0.69 ± 1.03	0.53 ± 0.80^bc^
Magnoliales	9	0.64 ± 1.40	0.63 ± 1.22	0.63 ± 1.26^bc^
Asterales	11	0.58 ± 0.42	1.11 ± 0.78	0.80 ± 0.60^c^
Degrees of freedom (DF)				13
F value				2.14
Pr > F				0.0114

The orders are arranged from highest to lowest activity (n = the number of tree species analysed in each order; mean MIC values followed by the same superscript letter do not differ significantly at the 5% confidence level).

The differences between the mean MIC’s of the orders against the Gram-negative bacteria ranged from 0.29 to 0.60 mg/ml (Table 3). The orders which had the highest activities were Myrtales and Fabales of which the mean MIC values were 0.29 and 0.30 mg/ml respectively. A *post-hoc* least square mean test (LSM) revealed that the activities of only a few orders differed significantly at the p < 0.05 level. The activities of the orders Myrtales and Fabales were significantly higher compared to the activities of Sapindales, Gentianales, Lamiales, Magnoliales, Asterales and Proteales. Even though the orders Malvales and Ericales had the third and fourth highest mean activities, their activities were only significantly higher (p < 0.05) than that of the Proteales. The orders Proteales and Asterales yielded the lowest mean activities against Gram-negative bacteria compared to the other orders. With a few exceptions there were not major differences in the mean activities of the orders against the two Gram-negative bacteria (Table 3).

**Table 3:** **Mean minimum inhibitory concentrations (MIC) ± standard deviation (SD) of the species present in the different orders against the Gram-negative bacteria**

Order	n (≥9)	Mean MIC (mg/ml)		
		***P. aeruginosa***	***E. coli***	Mean (Gram-negative bacteria)
Myrtales	25	0.28 ± 0.56	0.30 ± 0.59	0.29 ± 0.56^a^
Fabales	57	0.26 ± 0.55	0.34 ± 0.60	0.30 ± 0.51^ab^
Malvales	26	0.38 ± 0.73	0.31 ± 0.59	0.34 ± 0.64^abc^
Ericales	30	0.33 ± 0.77	0.36 ± 0.79	0.34 ± 0.75^abc^
Apiales	9	0.49 ± 0.66	0.26 ± 0.59	0.36 ± 0.53^abcd^
Rosales	28	0.37 ± 0.58	0.39 ± 0.58	0.38 ± 0.51^abcd^
Malpighiales	71	0.43 ± 0.66	0.37 ± 0.71	0.40 ± 0.62^acd^
Celastrales	19	0.39 ± 0.96	0.44 ± 0.48	0.41 ± 0.68^abcd^
Sapindales	64	0.45 ± 0.82	0.41 ± 0.84	0.43 ± 0.78^cd^
Gentianales	64	0.46 ± 0.75	0.43 ± 0.68	0.45 ± 0.67^cd^
Lamiales	35	0.52 ± 0.88	0.40 ± 0.36	0.46 ± 0.58^cd^
Magnoliales	9	0.58 ± 0.80	0.42 ± 0.43	0.49 ± 0.59^cd^
Asterales	11	0.44 ± 0.43	0.57 ± 0.82	0.50 ± 0.52^cd^
Proteales	28	0.46 ± 0.78	0.78 ± 0.67	0.60 ± 0.70^d^
Degrees of freedom (DF)				13
F value				2.27
PR > F				0.0065

The orders are arranged from highest to lowest activity (n = the number of tree species analysed in each order; mean MIC values followed by the same superscript letter do not differ significantly at the 5% confidence level).

The mean MIC’s of the orders against the fungi ranged from 0.28 to 0.59 mg/ml (Table 4). Among the orders, Malvales had the highest mean antifungal activity. An ANOVA performed on the data set followed by a *post-hoc* least square mean test (LSM) established that the activity was significantly higher (p < 0.05) compared to those recorded for the orders Gentianales, Sapindales, Ericales, Rosales and Celastrales. Members of the order Proteales yielded the second highest activity, but it was significantly higher (p < 0.05) compared to only the order with the lowest activity (Celastrales). The mean activity of the order Celastrales against the fungi was significantly lower (p < 0.05) compared to the activities of the orders Malvales, Proteales and Fabales. The order Rosales also had a relatively low mean activity but it was only significantly lower compared to Malvales. With a few exceptions there were not major differences in the mean activities of the orders against the fungi (Table 4).

**Table 4:** **Mean minimum inhibitory concentrations (MIC) ± standard deviation (SD) of the species present in the different orders against the fungal organisms**

Orders	n (≥9)	Mean MIC (mg/ml)		
		***C. albicans***	***C. neoformans***	Mean (Fungi)
Malvales	26	0.31 ± 0.76	0.25 ± 0.74	0.28 ± 0.68^a^
Proteales	27	0.38 ± 0.44	0.29 ± 0.75	0.33 ± 0.54^ab^
Fabales	56	0.37 ± 0.82	0.34 ± 0.90	0.35 ± 0.79^ab^
Apiales	9	0.41 ± 1.06	0.37 ± 0.94	0.35 ± 0.92^abc^
Magnoliales	9	0.36 ± 0.99	0.35 ± 1.09	0.36 ± 0.92^abc^
Malpighiales	71	0.36 ± 0.91	0.36 ± 0.86	0.36 ± 0.79^abc^
Myrtales	25	0.38 ± 1.06	0.41 ± 0.98	0.40 ± 0.97^abc^
Lamiales	35	0.42 ± 0.91	0.41 ± 1.01	0.42 ± 0.90^abc^
Gentianales	64	0.47 ± 1.02	0.38 ± 0.93	0.42 ± 0.90^bc^
Sapindales	63	0.44 ± 1.04	0.44 ± 0.97	0.44 ± 0.91^bc^
Ericales	30	0.55 ± 1.11	0.42 ± 0.77	0.48 ± 0.89^bc^
Asterales	11	0.48 ± 0.64	0.50 ± 0.94	0.49 ± 0.69^bc^
Rosales	28	0.57 ± 1.07	0.46 ± 0.94	0.51 ± 0.94^bc^
Celastrales	19	0.66 ± 0.71	0.53 ± 0.71	0.59 ± 0.59^c^
Degrees of freedom (DF)				13
F value				1.29
Pr > F				0.2149

The orders are arranged from highest to lowest activity (n = the number of tree species analysed in each order; mean MIC values followed by the same superscript letter do not differ significantly at the 5% confidence level).

To facilitate comparison of the activities of different orders against different pathogens classes, the mean MIC’s of the orders against each of the pathogen classes were ranked from high activity (low number) to low activity (high number) (Table 5). Species in the Fabales had relatively high activities against all pathogen classes while species in the Asterales had relatively low activities against all pathogen classes. The species in the Ericales and Myrtales had relatively high activities against both Gram-positive and Gram-negative bacteria.

**Table 5:** **Comparison of the ranking of activities (high activity = low MIC and low number) against each of the pathogen classes of species in the different orders**

Order	n (≥9)	Rank of activity		
		Gram-positive bacteria	Gram-negative bacteria	Fungi
Apiales	9	9	5	**3b**
Asterales	11	*14*	*13*	*12*
Celastrales	19	**1a**	8	*14*
Ericalis	30	**4b**	**3b**	*11*
Fabales	55	**4a**	**2**	**3a**
Gentianales	64	*11b*	*10a*	8b
Lamiales	35	*11a*	*10b*	8a
Magnoliales	9	*13*	*12*	5a
Malpighiales	71	7	7	5b
Malvales	26	8	**3a**	**1**
Myrtales	25	**1c**	**1**	7
Proteales	28	*10*	*14*	**2**
Rosales	28	**1b**	6	*13*
Sapindales	64	6	9	10

Orders with the highest and lowest activities in each class are identified (n = the number of tree species analysed in each order; four to five highest rankings are printed in bold; four to five lowest rankings are printed in italics; observations with equal MIC values were ranked with similar numbers but separated by ‘a’, ‘b’ and ‘c’).

There is evidence for some selectivity of activities. Species in the Proteales had very high antifungal but very low antibacterial activities. The same situation was present to a lesser degree in the species in the Apiales and Magnoliales. Species from the Rosales, Celastrales and Myrtales had a higher antibacterial than antifungal activity. Species in the Celastrales appeared to have higher activities against Gram-positive than Gram-negative bacteria. These species also had very high activities against the Gram-positive bacteria but had the lowest activities against the fungal organisms.

### Antimicrobial activities of tree species within orders with the highest and lowest mean antibacterial activities

**Figure 1:**
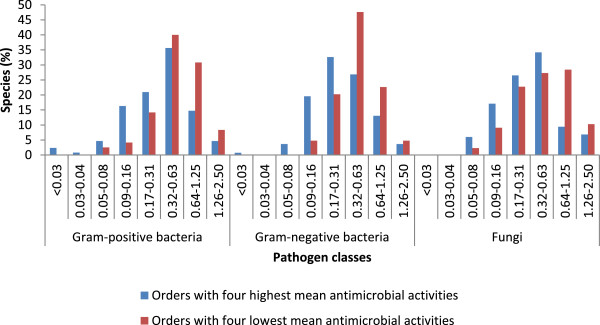
**The percentage of species inhibiting each of the pathogen classes across a range of MIC categories between orders with the four highest and four lowest mean activities indicating the distribution of activities.**

It is important to determine how useful mean antimicrobial activities of species in different orders are to predict activities of other species in that order. To determine the practical value of the differences between orders with high and low activity we compared the antimicrobial activities of all the tree species present in the four orders with the highest mean activities with the antimicrobial activities of all the tree species in the four orders with the lowest mean antimicrobial activities against the Gram-positive bacteria, Gram-negative bacteria and the fungi respectively (Figure 1).

Several authors consider that only MIC values of 0.1 mg/ml and lower represents good activity [25, 30–32]. In our serial dilution range we had results for 0.16 and 0.08 mg/ml. To facilitate statistical analyses and comparisons we considered that an MIC of 0.16 and lower would represent interesting activities. Against all pathogen classes there were a distribution of activities with some species in the high mean activity orders having very low activities and some species in the low mean activity having good activities.

Among the most promising orders against the Gram-positive bacteria, 24% of the species had interesting activities with mean MIC’s of 0.16 mg/ml and lower compared to 7% of the species in the orders with the lowest mean activities (Figure 1). This may mean that there is a three times better probability of finding extracts with high activity against Gram-positive bacteria from a tree species included in the high activity order than from one of the low activity orders.

Among the orders with the highest mean activities against the Gram-negative bacteria 24%, of the species had interesting activities (MIC ≤ 0.16 mg/ml) opposed to 5% of the species among the orders with the lowest mean activities (Figure 1). This may represent a fivefold higher probability of finding active extracts in these orders.

Among the orders with the highest activities against the fungal organisms, 23% of the species had interesting activities with MIC’s of 0.16 mg/ml and lower compared to 11% of the species in the orders with the least promising antifungal activities (Figure 1). This may represent a twofold higher probability of finding active extracts in these orders.

## Discussion

### Distribution of species in orders

The number of southern African species we collected from different tree orders varied widely due to classification systems that assemble unequal numbers of species in families and families in orders. These size differences complicated the comparisons of the mean MIC’s between the orders and only the orders of which we analysed nine or more species were compared since statistical analyses required a larger sample size. Only 14 of the 38 orders met this requirement because the range of order sizes was extremely skewed with far more small than large orders (Table 1). Despite this, the species in the 14 orders we analysed represented 89% of all the species investigated. Several of the 14 orders such as Asterales, Celastrales, Magnoliales and Proteales contained only one family although each family enclosed several southern African tree genera and species.

### Mean activities of species in different orders

Some of the orders had promising activities against more than one pathogen class which may indicate that members of these orders contain a broad spectrum of antimicrobials and may provide good leads (Table 5). The order Fabales, containing the Fabaceae and Polygalaceae families had relatively high mean activities against all three pathogen classes (Tables 2, 3, 4 and 5). The family Fabaceae contains most of the diversity in this order in the southern African region and is a family widely used in traditional medicine [52]. The family Polygalaceae makes up a much smaller proportion of the diversity of the family.

The order Celastrales was one of the orders with the highest activity against the Gram-positive bacteria (Table 2) and comprises of two tree families of which only representatives of one (Celastraceae) is found in the southern African region [39]. Therefore the activity of the order reflects that of a single family although several genera within the family were analysed. The family Celastraceae is one of the ten largest families in the region [39] and contains several tree genera of which 19 tree species were analysed in this study. It may be interesting to determine the antimicrobial activities of representatives of the two tree species of the other family Lepidobotryaceae, which naturally occurs in Central and East Africa as well as in South America.

The order Rosales had high antibacterial activities against Gram-positive bacteria (Tables 2 and 5) but had low activities against the fungi (Tables 4 and 5). This order comprises of six families in the southern African region [39] and representatives of all six were analysed. All three core families (Moraceae, Rhamnaceae and Rosaceae) in the order Rosales had very low activities against the fungi while all three core families had the highest activities compared to the other families against the Gram-positive bacteria [53]. Considering that fewer than three species per family were analysed within the other families (Cecropiaceae, Celtidaceae and Urticaceae), it should be worthwhile to investigate more species of the genera in these families against the Gram-positive bacteria.

The antibacterial activities of the orders Myrtales and to a lesser degree Ericales were ranked high against both Gram-positive and Gram-negative bacteria (Tables 2, 3 and 5), but was less active against the fungi (Tables 4 and 5). Ericales contained families such as Ebenaceae, Ericaceae, Myrsinaceae and Sapotaceae. The order Myrtales contained seven tree families and representatives of six were analysed. The Rhynchocalycaceae, a single genus family, was not collected. The families analysed were mostly small, consisting of a single tree genus, except for Combretaceae and Myrtaceae.

Some orders had low activities against more than one of the pathogen classes and members of these orders would be less promising candidates for further screening tests. For example, the order Asterales were ranked relatively low against all pathogen classes (Table 5). Although several species were analysed within this order, they are contained within a single family (Asterales).

The orders Gentianales and Lamiales had relatively low rankings against both Gram-positive and Gram-negative bacterial classes while the order Celastrales had a relatively low antifungal activity (Table 5). These orders may have fewer species with high activities against the specific pathogen class and may therefore be less promising candidates for future antimicrobial investigations and should probably not be prioritised in future screening programmes. Douwes *et al.* [54] investigated the frequency of species from different orders used by traditional healers in South Africa for treating various ailments based mainly on herbarium records. They identified seven “hot” plant orders such as Malpigiales, Fabales, Gentianales, Asterales, Solanales, Malvales and Sapindales that have significantly more taxa used ethnomedicinally. They also identified that species from the orders Rosales, Proteales, Poales, Asparagales and Caryphyllales were used less frequently than predicted based on the number of species. From their literature study and data mining, diverse bioactive compound were present in the plant families from the “hot” plant orders. They concluded that these taxa are selected traditionally on the basis of bioactivity, which is reflected in chemical diversity. The orders they identified as frequently used does not coincide with our high activity orders with the exception of Fabales and to a degree the Proteales as a low activity order for the bacteria. The lack of agreement is interesting but may not be surprising because we investigated antimicrobial activity of acetone leaf extracts of trees and they investigated the listed use of mainly water extracts of all plants of different ailments. It is also not clear if their list derived from Arnold *et al.* [20] included species used for magical properties.

### Selective activity in orders

A number of orders had relatively high activities against one of the pathogen classes while their activities were notably lower against other pathogen classes which could indicate a selective activity (Table 5). A species with selective activity may indicate that the activity is not due to a general metabolic toxin that may also be toxic to mammalian cells. Orders such as Malvales and Apiales had higher activities against the Gram-negative bacteria and the fungi (Tables 3, 4 and 5) compared to the Gram-positive bacteria (Tables 2 and 5).

It is interesting that the order Proteales had high activities against the fungi and much lower activities against the bacteria, especially against the Gram-negative bacteria. The tree species analysed within Proteales are contained within a single family (Proteales).

### Predictive value of the results

The high variability complicates the identification of superior plant orders against a given pathogen class because the orders contain species with varying degrees of antimicrobial activity. Therefore, we compared the activities of the tree species analysed within the four most promising orders with those of the four least promising orders.

Among the four most promising orders and the orders with the four least promising activities (Figure 1), a number of species had interesting activities (≤ 0.16 mg/ml) while others had very low mean activities (≥ 1.25 mg/ml). The results indicate that potentially useful species (≤ 0.16 mg/ml) were found in both groups, although fewer were found in the less promising orders. It appears that there is a normal distribution of activity within orders (Figure 1), but that the means of orders are close and that there is substantial intersection across all orders. The variation within the orders may be an indication of the large diversity of secondary metabolites in plants, even in closely related species.

Although the high intra-taxa variation means taxa with a low mean activity may contain species with a high antimicrobial activity and *vice versa* these results indicate that there is a threefold higher probability of finding species with interesting activity against Gram-positive bacteria from orders with high mean activity than from orders with a low mean activity.

With Gram-negative bacteria, there is a fivefold higher probability of finding species with interesting activity from orders with high mean activity than from orders with a low mean activity. The probability is two times higher to find species with interesting activity against fungi from orders with high mean activity than from orders with a low mean activity.

The probability of finding species with higher activities may be higher if species from the order with the highest mean activity was compared with species from the order with the lowest mean activity. By analysing the antimicrobial activity on a family basis, lower variation and higher predictive value may be obtained.

## Conclusions

The results indicate that there is a correlation between the taxonomy at the order level and antimicrobial activity. Despite the very high variation in antimicrobial activities within orders there were statistically significant differences between orders of which we analysed more than eight species.

Because species in the promising orders have a higher probability of yielding extracts with an interesting antimicrobial activity these orders represents priority areas for further antimicrobial research. This could maximise the number of leads that are found in the screens in a shorter time than random collections.

Manuscripts are in preparation to compare the antimicrobial activities at family level which could lessen the intra-taxa variation because the groups are smaller as well as at genus level. In some cases we may be able to predict with a higher confidence which families or genera could facilitate the discovery of plant extracts that can be used to combat microbial infections in animals and humans [53].

To determine the potential value of extracts to control infections in humans and animals it is important that the cellular toxicity should be determined. An extract with a high selectivity index, i.e. a high safety and a lower antimicrobial activity would be preferential to an extract with high antimicrobial activity accompanied by a high cellular toxicity against mammalian cells. We are in the process of determining the cellular toxicity of extracts with high antimicrobial activity.
